# Tolerability and Efficacy of Memantine as Add on Therapy in Patients with Migraine

**Published:** 2017

**Authors:** Farhad Assarzadegan, Mohammad Sistanizad

**Affiliations:** a *Department of Neurology, Emam Hossein Medical and Educational Center, Shahid Beheshti University of Medical Sciences, Tehran, Iran.*; b *Department of Clinical Pharmacy, Faculty of pharmacy, Shahid Beheshti University of Medical Sciences, Tehran, Iran .*; c *Department of Pharmaceutical Care Unit, Emam Hossein Medical and Educational Center, Shahid Beheshti University of Medical Sciences, Tehran, Iran.*

**Keywords:** Headache, Migraine, Memantine, Refractory

## Abstract

Prophylactic migraine treatment has always been a challenge. Efficacy and tolerability are two main issues in current approved migraine prevention regimens. Since some migraine patients fail approved preventative agents, experts are always seeking newer agents. Memantine, a glutaminergic antagonist, could potentially be one of these agents. Objective of current study is assessing the efficacy of memantine as a preventative migraine treatment and its potential side effects.

In this study, 127 migraine patients meeting the criteria for starting preventative therapy (> 4 headache days/month) are included in the study. All patients were previously failed in at least one trial of adequate preventive therapy. After a 30 day baseline observation, patients started memantine for 3 months, beginning at 5 mg/day, which increased by 5 mg/week up to a maximum of 20 mg a day if symptoms did not improve. Headache frequency, duration, and severity were assessed at the end of the treatment phase. 102 patients completed the study.

In the study population, headache frequency reduced from 9.9 days/month at baseline to 5 days/month at 3 months (P < .001). The mean severe pain reduced from 6.9 to 3.6 at 3 months (P < .001). Headache duration significantly reduced at 3 months, compared with baseline (P < .001). Side effects related to memantine consumption were uncommon and generally mild.

Based on preliminary data, there is some evidence that memantine might be useful in the treatment of refractory migraine. This is in line with previous pilot and open label studies. However, double blind studies are still needed.

## Introduction

Headaches is the most common outpatient neurological complain. Migraine is a common primary headache disorder that affects people of a variety of ages and sometime could be debilitating. Many migraineurs who seek care in headache clinics are refractory to treatment (1, 2) . Refractoriness is defined based on the chronicity, frequency, and severity of the headaches, as well as on subjects experiencing less than expected benefit from standard therapies. Defining refractory migraine has been the subject of a great deal of interest (3-5).

**Figure 1:**
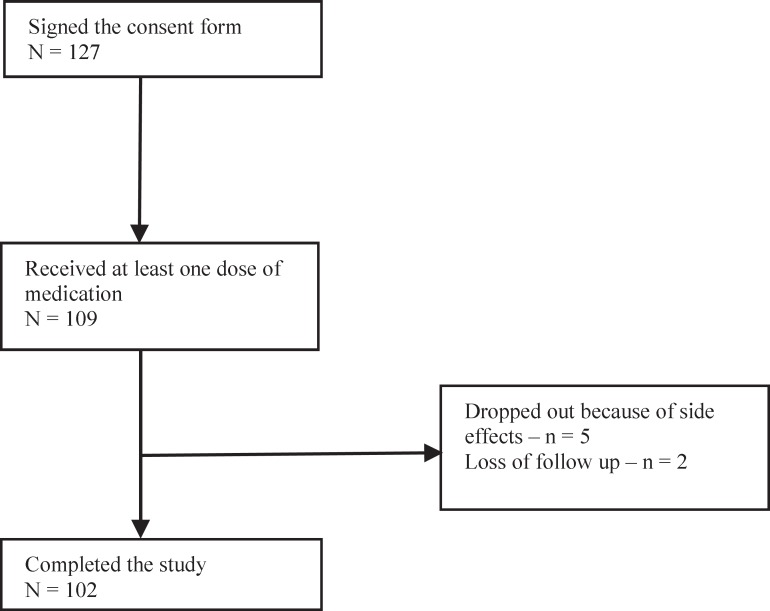
Flowchart of enrollment and participation

**Table-1:** Headache location and symptoms reported during 1 month baseline observation in 109 patients with diagnosis of chronic refractory migraine

**Symptoms**	**Number Patients (%)**
Headache** (**Bilateral)	79 (77.4%)
Headache (Unilateral)	23 (22.6%)
Photophobia	82 (80.4%)
Phonophobia	82(80.4%)
Nausea	71(69.6%)
Vomiting	22(21.5%)
Osmophobia	61(59.8%)
Blurred vision	18(17.6%)
Autonomic	11(10.8%)
**Headache location**	
Frontal	71 (69.6%)
Temporal	27 (26.5%)
Vertex	36 (34.6%)
Occipital	33 (32.3%)

**Table 2:** Assessment of endpoints in 102 patients who completed 3 month memantine treatment

**Endpoint**		**Baseline**	**After 3 Months**	***P *** **value**
Number of headache mean±SD		9.9±8	5 ±6.8	0.001
Severity of headaches, mean±SD		6.9±1.6	3.6±1.5	0.001
Length of headache, n (%)	<4 hours	9 (8.8)	60 (58.8)	0.001
	4-24 h	36 (32.3)	36 (32.3)	
	>24 h	57 (55.90	6 (5.9)	

Despite the existence of multiple well established migraine preventive therapies, there is a significant proportion of refractory migraine patients for whom currently available therapies are either ineffective or poorly tolerated. Most preventive agents used in this context have not been examined specifically for the treatment of this syndrome (6-8). Medications for refractory migraine are used empirically based on their efficacy in the treatment of episodic migraine (6, 7), and patients are often treated with multiple drugs (9). As a consequence, side effects, poor compliance, and disappointing outcomes are common. Cognitive symptoms also emerge frequently in this context, either as a consequence of chronic pain (10, 11) or of the medications used to treat refractory migraine (12, 13). 

Migraine is increasingly viewed as an episodic disorder of brain excitability. Glutamate, is an excitatory neurotransmitter in Central Nervous System mentioned prominently in theories of migraine pathophysiology (14). Signaling by glutamate, the primary excitatory neurotransmitter in the central nervous system, is therefore an appealing target for migraine therapy. N-methyl-D-aspartate (NMDA) receptor antagonists are known to inhibit cortical spreading depression, which is believed to be a fundamental mechanism of migraine (15, 16). 

NMDA receptor antagonists block neurotoxicity induced by excessive glutamate release into the synaptic cleft. However, clinical trials of a large number NMDA receptor antagonists (mainly for stroke) have failed due to the side effects resulting from the blockade of normal neuronal function (17, 18). 

Contrasting with the more potent NMDA receptor antagonists, memantine, an uncompetitive, low-affinity, open channel blocker, is clinically well tolerated (19-21). This safe clinical profile seems to result from its “use-dependent” prevention of hyperactivity of the NMDA receptor channel complex without disrupting normal activity (18, 20).

Memantine is the first in a novel class of Alzheimer’s disease medications acting on the glutaminergic system. Memantine is a moderate-affinity voltage-dependent noncompetitive antagonist at glutaminergic NMDA receptors. By binding to the NMDA receptor with a higher affinity than magnesium (Mg2+) ions, memantine is able to inhibit the prolonged influx of calcium (Ca2+) ions associated with neuronal excitotoxicity (16). Glutamate is hypothesized to be important in migraine pathophysiology (14), and individuals with refractory migraine frequently complain of cognitive problems, either as a function of the disease itself or as a side effect of medications. We conducted a study to prospectively assess the efficacy and tolerability of memantine in the treatment of patients with chronic disease who referred to Imam Hossein Hospital and the majority of them had failed standard acute and preventive therapy.

## Materials and Methods

This was a prospective, open-label trial conducted in the headache clinic of Imam Hossein Hospital from 2011 to 2013. A total of 127 participants, male and female, aged 25 to 50, were enrolled consecutively when attending the headache clinic for scheduled visits and the majority of them had failed to the previous preventive therapy for migraine.

The potential participants were asked for a written informed consent. The study as well as all the forms and questions to be asked were approved by the Ethical Committee of Shahid Beheshti University of Medical Sciences. Inclusion and exclusion criteria were:

**1.** Refractory migraine, defined as follow:

A. At least one of I or II:

I. Episodic migraine with headaches happening on 8-14 days per month in the last 3 month.

II. Transformed migraine according to the criteria proposed by Silberstein and Lipton ^(22)^.

B. Previous failure to at least one standard migraine preventive medication, used in adequate doses for at least 3 months.

**2.** No previous use of memantine.

**3.** No major depression.

The baseline observation period consisted of one month, and information including age, sex, having or not having aura, disease symptoms and location of headache, experience of taking sedatives, and the results of neurologic examinations were collected before treatment. 

After the baseline period, memantine administration started. Trial doses were prescribed individually. All patients started with 5 mg/day. Every 2 weeks for the participant with no side effects reporting a feeling of not having complete satisfaction, 5 mg was added up to a maximum amount was 20 milligrams a day. Patients were maintained at the dose at which they were satisfied with the reduction in headache frequency. The treatment lasted for 3 months. Patients were clinically reevaluated in person every two weeks. In case of intolerance or adverse effects the dose was reduced or the patient was excluded from the trial.

The primary endpoint was number of headache days (headache frequency) after 3 months treatment, as compared with the baseline period. Secondary endpoint was the headache severity, which measured by the linear-optical criterion which is rated from 0 (without headache) to 10 (severe headache). Other endpoint included length of headache which was based on less than 4 h, 2 to 24 h and more than 24 h. 

Data were collected by a single physician using standard clinical forms and interview questions. The quantitative data were presented as mean + SD and qualitative ones were expressed in frequency. The analysis was done by SPSS (version 17.0, Chicago, IL, USA) based on paired t-test and Wilcoxon comparison betwwen the before and after endpoints. P value less than 0.05 was considered a significant level. 

## Results

Of 127 subjects enrolled, 109 completed the one month baseline assessment and started with memantine treatment. The study population consisted of 77.4% females, mean age of 38.9 (SD = 11.4) years) and 22.6% males, and mean age of 41.1years (SD = 10.6). A total of 102 (93.6%) subjects completed the full treatment period; 5 (4.6%) dropped out the study because of side effects and 2 (1.8%) were lost to complete the full treatment period (Figure 1)

During the one month of baseline observation, 81 (74.3 %) of participants had at least one episode of migraine with aura, while the remaining had migraine without aura. All subjects were using other preventive medications. A total of 44 (40.4 %) were using one preventive drug, while 59.6 % were using 2 or more. They were on stable doses of medication for a minimum of 3 months (Mean = 4.1, SD = 1.3). The neurologic examinations were normal in all patients. 

In the baseline, 77.4% of the refractory migraine patients complained about bilateral headache and 22.6% complained about unilateral headache (table-1). Photophobia, phonophobia, and nausea were the most prevalence symptoms in patients. 7 patients had impairment in daily functioning. Location of pain in patients is shown in Table-1. Some patients complained about more than one pain location in head.

After 3 month treatment with memantine, headache severity in patients significantly reduced to 3.6 ± 1.5from 6.9 ± 1.16 in the baseline (p < 0.001) (Table 2). 

The frequency of having headache per month was 9.9 ± 0.8 before intervention and 5.6 ± 0.8 after intervention for three month taking memantine. This reduction was significant (p < 0.001) (Table 2). 

Furthermore, the length of headache in each migraine attack was significantly lower after treatment. 60 patients (58.8%) reported pain episodes of less than 4 h, compared to before treatment when only 9 patients reported migraines lasting less than 4 h (8.8%). 57 patients (55.9%) complained about headaches more than 24 h in the baseline which reduced to only 6 patients after intervention (5.9%). The reported reduction in frequency and severity after treatment were statistically significant (p < 0.001) (Table-2).

## Discussion

Our results suggest that memantine could be an effective therapy for prevention of refractory migraine in patients in whom other established migraine preventive therapies have failed. The patients in this study had a high baseline headache frequency, and most had tried at least two standard migraine preventive therapies. Even in this refractory migraine population, the majority of patients had a significant reduction in headache frequency, length, and severity. Memantine was generally well tolerated, although there were side effects resulted in discontinuation of the medication in approximately 4.6% of patients. Our results are in consistent with previous preliminary studies and the reports have shown an effect of memantine in migraine (23, 24). We concur with the dosages used by Bigal *et al.* (23), who also used 10-20 mg/day in order to achieve reduction from a mean of headache days per month after 3 months (P < .01) in patients already under prevention with one or more drugs. Charles et al., found that treatment for two months with memantine is in agreement with our results (24). 

Migraine pain-relay centers, including the trigeminal ganglion, trigeminal nucleus caudalis, and thalamus contain glutamate-positive neurons (25, 26). Therefore, the link between the glutaminergic system and migraine should be considered. The glutaminergic system is frequently mentioned as a possible core system for the initiation of cortical spreading depression and trigeminal vascular activation (14). In addition, the central sensitization and the transformation of episodic into chronic migraine are mediated, at least partially, by excessive glutaminergic activation (27). So, Glutamate receptor-subtype antagonists are effective in preclinical models of migraine. It has been suggested that chronic pain can be maintained by a state of sensitization within the central nervous system that is mediated in part by glutamate and aspartate binding to the NMDA receptor. A number of antagonists to the NMDA receptor like memantine is antinociceptive in animal models but associated with significant dose-limiting side effects (25, 26). 

It may be speculated that memantine could be able to compete with magnesium and inhibit the prolonged influx of calcium, therefore avoiding the neuronal excitotoxicity. This makes it even more attractive for migraine prevention as been already suggested (23, 27).

Previous studies suggest that memantine preferentially blocks excessive NMDA receptor activity without disrupting normal activity. memantine does this through a noncompetitive, low-affinity, open-channel blocker; it enters the receptor-associated ion channel preferentially when it is excessively open, and, most importantly, its off-rate is relatively fast so that it does not substantially accumulate in the channel to interfere with subsequent normal synaptic transmission (15) . It is therefore possible that memantine could reduce episodic increase in cortical excitability underlying migraine. This potential mechanism is supported by our observation that memantine reduced the frequency of headache. Finally and as recently described by means of functional magnetic resonance imaging and magnetencephalography, memantine induces cortical reorganization. This normalization of cortical function seems to be associated with decreased pain levels in cases of complex regional pain syndrome (28). A similar effect of memantine in migraine could be assumed.

In this open-label; exploratory trial, we found that memantine was well tolerated. However, several precautions should be considered when analyzing the implications of our results. First, although this is a prospective study, it is not controlled by placebo. It is an open-label study and is limited by lack of blinding. There are multiple biases that could therefore confound these observations, and there could be a highly significant placebo effect. Second, we included refractory migraine with and without medication overuse. Those overusing medication had failed detoxification protocols in the past. No patient stopped medication overuse during our study. Because studies show that preventive medication is often ineffective in patients overusing acute medication, we may have underestimated the benefits of memantine by including overusers. Nonetheless, the results are encouraging because of the patient population that was studied, the overall tolerability of the medication, and the scientific rationale for a mechanism of action in migraine prevention. 

## Conclusion

The findings of this study indicate that memantine could potentially be a promising drug for the treatment of refractory migraine, although it has neither been submitted to nor approved by the Food and Drug Administration for this use. 

The results of our study are in line with previous pilot and open label studies (22).

A necessary future steps forward is to conduct a double-blind placebo-controlled study of memantine in the treatment of refractory migraine.
